# Correlation of visual field deficits and q-ball high-resolution fiber tractography of the optic radiation for adjacently located intracerebral lesions: preliminary results from a single-center prospective study

**DOI:** 10.1007/s10143-023-02278-9

**Published:** 2024-01-05

**Authors:** Pavlina Lenga, Moritz Scherer, Robin Peretzke, Peter Neher, Jessica Jesser, Christina Beisse, Andreas W. Unterberg, Becker Daniela

**Affiliations:** 1 Department of Neurosurgery, Heidelberg University Hospital, Heidelberg, Germany; 2 German Cancer Research Center, Division of Medical Image Computing, Heidelberg, Germany; 3 Department of Ophthalmology, Heidelberg University Hospital, Heidelberg, Germany; 4 Department of Radiation Oncology, Heidelberg University Hospital, Heidelberg, Germany; 5 IU International University of Applied Sciences, University of Applied Sciences, Mannheim, Germany

**Keywords:** Fiber tracking, Diffusion tensor imaging, Q-ball imaging, High-resolution fiber tractography, Optic radiation, Visual field deficits

## Abstract

Visual field deficits (VFDs) are common in patients with temporal and occipital lobe lesions. Diffusion tensor fiber tractography (DTI-FT) is widely used for surgery planning to reduce VFDs. Q-ball high-resolution fiber tractography (QBI-HRFT) improves upon DTI. This study aims to evaluate the effectiveness of DTI-FT and QBI-HRFT for surgery planning near the optic radiation (OR) as well as the correlation between VFDs, the nearest distance from the lesion to the OR fiber bundle (nD-LOR), and the lesion volume (LV). This ongoing prospective clinical trial collects clinical and imaging data of patients with lesions in deterrent areas. The present subanalysis included eight patients with gliomas near the OR. Probabilistic HRFT based on QBI-FT and conventional DTI-FT were performed for OR reconstruction based on a standard diffusion-weighted magnetic resonance imaging sequence in clinical use. Quantitative analysis was used to evaluate the lesion volume (LV) and nD-LOR. VFDs were determined based on standardized automated perimetry. We included eight patients (mean age 51.7 years [standard deviation (SD) 9.5]) with lesions near the OR. Among them, five, two, and one patients had temporodorsal, occipital, and temporal lesions, respectively. Four patients had normal vision preoperatively, while four patients had preexisting VFD. QBI-FT analysis indicated that patients with VFD exhibited a significantly smaller median nD-LOR (mean, −4.5; range −7.0; −2.3) than patients without VFD (mean, 7.4; range −4.3; 27.2) (*p* = 0.050). There was a trend towards a correlation between tumor volume and nD-LOR when QBI-FT was used (rs = −0.6; *p* = 0.056). A meticulous classification of the spatial relationship between the lesions and OR according to DTI-FT and QBI-FT was performed. The results indicated that the most prevalent orientations were the FT bundles located laterally and intrinsically in relation to the tumor. Compared with conventional DTI-FT, QBI-FT suggests reliable and more accurate results when correlated to preoperative VFDs and might be preferred for preoperative planning and intraoperative use of nearby lesions, particularly for those with larger volumes. A detailed analysis of localization, surgical approach together with QBI-FT and DTI-FT could reduce postoperative morbidity regarding VFDs. The display of HRFT techniques intraoperatively within the navigation system should be pursued for this issue.

## Introduction

The optic radiation (OR) is crucially involved in visual pathways. Performing neurosurgery near the OR, which comprises the lateral temporal, inferior parietal, and occipital regions, is challenging given the risk of postoperative visual field deficits (VFDs). The prevalence of VFDs resulting from OR injuries ranges from 20 to 100%, which has been observed in cases of epilepsy surgeries [14, 15]. This high morbidity rate can be attributed to the anatomy of the OR, which originates from the lateral geniculate nucleus (LGN) and traverses the temporal stem before terminating at the calcarine sulcus [24].

The Meyer loop (ML) is a critical segment of the OR and is located in the anterior-most portion. It carries visual information from the bilateral superior visual field. In this way, damage to the ML can cause superior quadrantanopia. Postoperative complications can be minimized by preserving key white matter pathways involved in large-scale networks. Accordingly, there has been increasing attention on diffusion-weighted magnetic resonance imaging (MRI)-based fiber tractography (FT) since it non-invasively facilitates maximal tumor resection while preserving neurological function, which improves survival and quality of life [27]. Specifically, diffusion tensor imaging (DTI)-based FT allows identification and visualization of functional sites at the cortical level as well as the trajectory of important fascicles within the white matter [3, 22]. Although this approach is user-friendly and already integrated into commonly used navigation systems, it has limited accuracy in the determination of the origin and cortical termination of fibers within the white matter, particularly when diffusion properties are disturbed in afflicted brain tissue. Recently, advanced FT techniques, based on high-angular-resolution diffusion imaging (HARDI) signals, have been proposed to address the limitations of DTI, known as high-resolution fiber tractography (HRFT). However, the clinical utility of techniques remains unclear given the frequently longer acquisition times required due to the larger number of encoding gradients or sophisticated and time-consuming postprocessing processes [2, 20, 26]. One already well-known technique for HRFT is q-ball imaging, which we already investigated for clinical applications so far and which has proven to be applicable [1, 5, 8].

Currently, the feasibility and efficacy for surgery of FT and HRFT in patients with lesions near the OR remain unclear. Among patients with epilepsy undergoing temporal lobe surgery, DTI-FT has been found to significantly reduce the incidence of postoperative VFDs [7]. However, in the context of neuro-oncology, the extent to which these techniques can mitigate the occurrence of postoperative VFDs remains unclear and has to be determined in the context as gross-total resection remains standard to extend patient survival.

Accordingly, we aimed to (1) assess the feasibility of using DTI and q-ball-based HRFT in presurgical planning for patients with lesions near the OR, (2) examine the relationship of the fiber tracking results with preoperative VFD, and (3) examine the relationship between detailed lesion localization and visual field conditions.

## Methods

### Study design, inclusion criteria, and exclusion criteria

Clinical and imaging data were prospectively collected and analyzed between April 2021 and February 2022. This study was conducted in accordance with the Declaration of Helsinki and approved by the local ethics committee (S-146/2017; S147/2017). Each patient provided written informed consent for study participation. This study included patients aged ≥ 18 years with gliomas near the OR whose preoperative MRI protocol included DTI sequences by default. The exclusion criteria included lesion located > 20 mm from the estimated fiber bundles, age < 18 years, and incomplete MRI data. One cavernoma in the proximity of the OR was excluded aiming to preserve the homogeneity of our patient cohort.

### Imaging data

For the objectives of our study, QBI was chosen due to its compatibility with the MITK platform, which is known for its user-friendly interface and suitability for potential intraoperative application. MITK offers a streamlined process for QBI that is conducive to the clinical environment where time efficiency and simplicity of use are paramount. The consideration for the clinical applicability of the technique guided our selection process. While other more complex techniques offer depth and sophistication, they often demand extensive computational resources and expertise in programming languages such as Python for the reconstruction of ODFs. For instance, the use of spherical deconvolution requires intricate postprocessing and model assumptions that extend beyond the immediate intraoperative timeframe. Similarly, compressed sensing techniques, while powerful in data reconstruction, necessitate advanced algorithmic implementations and substantial processing time, which may not be feasible in an intraoperative setting. Our goal was to implement a method that balanced technical capability with practicality in a surgical environment. Hence, the decision to utilize QBI was made not only based on its analytical robustness but also considering the operational constraints and the necessity for real-time processing capabilities in neurosurgical planning and intervention.

Up to 3 days before surgery, a preoperative MRI dataset was obtained using a 3 Tesla scanner (Magnetom Prisma, Siemens, Erlangen, Germany). This study used the following sequences: T1-weighted three-dimensional (3D) magnetization-prepared rapid gradient-echo (MPRAGE) with gadolinium, with a repetition time (TR) of 1790 ms, an echo time (TE) of 3.7 ms, a field of view (FoV) of 250 mm, 160 slices (slice thickness 1 mm) acquired in the sagittal plane, and an acquisition duration of 3 min and 29 s; a fluid-attenuated inversion recovery sequence with a TR of 8500 ms, a TE of 136 ms, a FoV of 230 mm, 25 slices, and an acquisition duration of 2 min and 52 s; a diffusion-weighted imaging sequence with a TR of 6600 ms, a TE of 87 ms, a FoV of 256 mm, 56 slices, utilizing a number of excitations I, *b* value of 1000 s/mm^2^, 64 non-collinear diffusion-encoding gradients, voxel size of 2×2×2 mm^3^, and an acquisition duration of 7 min and 50 s. The overall duration of the MRI session was approximately 25 min together with other routinely obtained MRI data.

The Medical Imaging Interaction Toolkit’s (MITK) open-source software MITK Diffusion (available at https://github.com/MIC-DKFZ/MITK-Diffusion) was used for fiber tracking (source: Medical Imaging Interaction Toolkit: MITK Diffusion Imaging). DTI and QBI tracking approaches used the same DW-MRI sequence. After the data were imported into MITK Diffusion, rigid registration was performed between the T1-MPRAGE and DW images. Subsequent preprocessing of the DW images involved head motion correction and eddy current correction through affine registration to the unweighted volume [11]. Subsequently, the tensors for DTI were calculated using the Insight Toolkit, respectively. For QBI, a diffusion orientation distribution function (dODF) was calculated. A spherical harmonics reconstruction with solid angle consideration was applied [1]. An order of 6 and otherwise default parametrization was defined for the spherical harmonics, enabling a reconstruction from 64 diffusion-encoding gradients.

FT for both DTI- and QBI-based fiber reconstructions was performed with a probabilistic approach. Three-dimensional regions of interest (ROIs) were manually segmented, one around the LGN and another over the visual cortex (Brodmann areas 17–19). False-positive fibers were excluded from the analysis using exclude ROIs.

Tractography parameters were set based on common recommendations and comparability. For QBI, the parameters included sharpening of the orientation distribution functions [8], a GFA cutoff of 0.15, step size of 0.5 voxels, angular threshold of 20, and minimum tract length of 20 mm. For DTI, the parameters included an FA cutoff of 0.15, step size of 0.5 voxels, angular threshold of 20°, and a minimum tract length of 20 mm [4].

High-grade gliomas (HGGs) were segmented according to T1-weighted sequences (T1-w) after administration of gadolinium contrast agent (Gd), while non-enhancing tumors (low-grade gliomas, LGGs) were segmented on fluid-attenuated inversion recovery (FLAIR) sequences. The segmentation of the relevant anatomical structures was meticulously conducted using Brainlab software. This process involved manual segmentation across all MRI slices, ensuring precise delineation of the targeted areas and facilitating accurate analysis and interpretation of our results.

### Procedures

To determine the nD-LOR, automatic calculation based on the 3D Euclidean distance transform algorithm was applied. In particular, the computation of the distance between the tract and tumor involves utilizing the “MitkDistanceToSegmentation” command within the MitkDiffusion tool. This algorithm determines the minimum distance from the tract’s envelope to the tumor segmentation.

Patients underwent automated full-field static 30-degree perimetry with *Octopus 900* (Haag Streit, Germany) at admission and before discharge.

The results were independently assessed by two seasoned ophthalmologists. The relationship between visual field defects (VFDs) and the anatomical location of the lesion was explored, specifically in terms of laterality and the spatial relationship between the optic radiation (OR) and the lesion. A defect depth less than −5 dB was defined as a visual field defect. This parameter was meticulously determined for each eye and quadrant individually. VFDs were subsequently stratified into three categories: “unchanged,” “deterioration” (inclusive of any deepening in defect depth), and “improvement,” by conducting a comparative analysis of preoperative and postoperative perimetry results. Consideration was given to patient compliance, which was quantified by the number of false-positive and false-negative responses.

### Statistical analysis

For categorical variables, frequency counts are presented as percentages. Continuous variables are presented as medians ± interquartile range [IQR] or mean and standard deviation (SD). Normality of data distribution was tested using the Shapiro-Wilk test. Univariate analysis was used for between-group comparisons (patients with vs. without VFD). Correlations were tested using Spearman’s correlation coefficients. Statistical significance was set at *p* = 0.05. All statistical analyses were performed using Statistical Package for the Social Sciences version 24.0.0.0 (IBM Corp., Armonk, NY, USA).

## Results

**Fig. 1:**
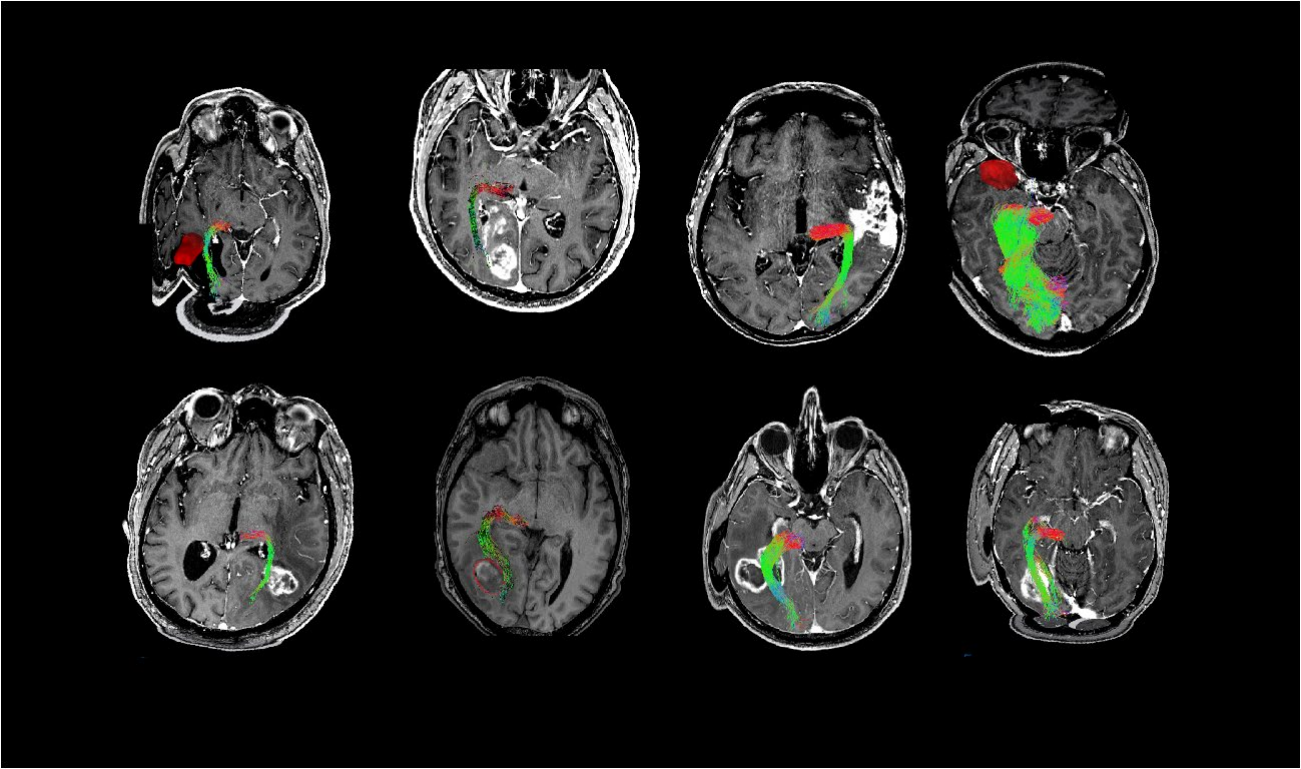
Overview of lesions and FT results (in numerical order). All MRI T1-weighted sequences, oblique view. FT all according to QBI. For patient 4, overlay of DTI-FT (pink) and QBI-FT (purple). MRI = magnetic resonance imaging; FT = fiber tractography; QBI = q-ball imaging; DTI = diffusion tensor imaging

This study included eight patients (mean age 51.7 [9.5] years) with lesions near the OR. Among them, eight patient had gliomas, near the OR. The mean postprocessing time for OR visualization in DTI and QBI using *MITK-Diffusion* was 21.5 min (SD 4.2) and 32.8 min (SD 7.9), respectively. DTI did not yield fibers in two cases, despite equal adjustments, parameters, and ROIs. In these cases, tumors differed in size, however, were high-grade gliomas with the typical contrast enhancement and had a large peritumoral edema. All lesions were resected completely, according to (early) postoperative MRI. Lesions and QBI-FT results are depicted in Fig. 1.

The localization category of the lesions was carried out in alignment with the methodology presented by Faust et al. [10], as detailed in Table 1. Specifically, we categorized the lesions according to our DTI-FT and HRFT results, respectively. The corresponding ophthalmological expert’s analysis is given: “equal = 0, deterioration = −1, improvement = 1.” Lesion localization was considered as “intrinsic” for DTI-FT, or HRFT, when nD-LOR showed negative values. Altogether, we see that a deterioration of the visual field is a common finding (7/8 cases). Notably, an equal or improved VF occurred for tumors in the medial temporal lobe and temporo-polar (patients 5 and 10). Deteriorated VF was seen for temporodorsal or occipital lesions, categorized as lateral and/or intrinsic according to FT.

**Table 1:** Classification of the lesions adapted for DTI-FT and QBI-FT with the methodology presented by Faust et al

Patient/anatomical localization	Localization by QBI-FT	Localization by DTI-FT	Histopathological findings	VF before surgery	VF postsurgery	VFD analysis
Temporodorsal lateral	Lateral	Lateral	Oligodendroglioma WHO 2, IDH 1 positive, 1p 19q co-deletion	Normal	Lower left quadrant	−1
Occipitomedian	Intrinsic	Intrinsic	Glioblastoma WHO 4, IDH negative, MGMT methylated	Right-sided hemianopsia	Central worsening	−1 (central defect)
Temporal	Intrinsic	Lateral	Glioblastoma WHO 4, IDH negative, MGMT methylated	Left-sided hemianopsia	Improvement in defect depth + in both upper and lower quadrants	1
Temporopolar	Polar	Polar	Astrocytoma WHO II, IDH negative	Normal	Normal	0
Occipitolateral	Lateral	Lateral	Glioblastoma WHO 4, IDH negative, MGMT methylated	Right-sided hemianopsia	Central worsening	−1 (central defect)
Temporodorsal lateral	Lateral	–	Glioblastoma WHO 4, IDH wildtype, MGMT not methylated	Left-sided hemianopsia	Worsening of defect depth	−1 (defect depth)
Temporodorsal lateral	Intrinsic	Intrinsic	Glioblastoma WHO 4, IDH wildtype, MGMT methylated	Normal	Left-sided homonymous hemianopsia	−1
Temporodorsal lateral	Intrinsic	–	Glioblastoma WHO 4, IDH wildtype, MGMT not methylated	Normal	Quadrantanopia	−1

Equal = 0, deterioration = −1, improvement = 1

### Correlation to visual field data

Four patients had normal vision and four presented with preexisting VFD. QBI-FT analysis indicated that patients with VFD exhibited a smaller median nD-LOR (mean −4.5; range −7.0; −2.3) than patients without VFD (mean 7.4; range −4.3; 27.2; *p* = 0.050), albeit these findings were not statistically significant. DTI-FT analysis revealed no significant between-group differences in nD-LOR. Patients with VFD had a non-significantly larger tumor volume than patients without VFD (mean 28.1, range 17.6; 39.1 vs. mean 11.1, range 5.5; 18.3; *p* = 0.09). These findings are summarized in Table 2. There was no significant correlation between tumor volume and nD-LOR calculated using DTI-FT; however, there was a correlation trend for a smaller nD-LOR analyzed using QBI-FT (rs = −0.6; *p* = 0.055).

**Table 2:** Comparative analysis of patients with and without visual field defects (VFDs) regarding tumor location and minimum tumor distance determined by fiber tracking (FT) techniques

	Normal VF (*n* = 4)	VFD (*n* = 4)	*p*
Nearest distance by QBI mean (range) (mm)	7.4 (−4.3; 27.2)	−4.5 (−7.0; −2.3)	**0.050**
Nearest distance by DTI, mean (range) (mm)	7.0 (−0.2; 20.0)	−1.4 (−3.9; 1.2)	0.165
Volume, mean (range) (cm^3^)	11.1 (5.5; 18.3)	28.1 (17.6; 39.1)	0.077

Bold *p* values indicate significant results

### Illustrative cases

#### Case 1

**Fig. 2:**
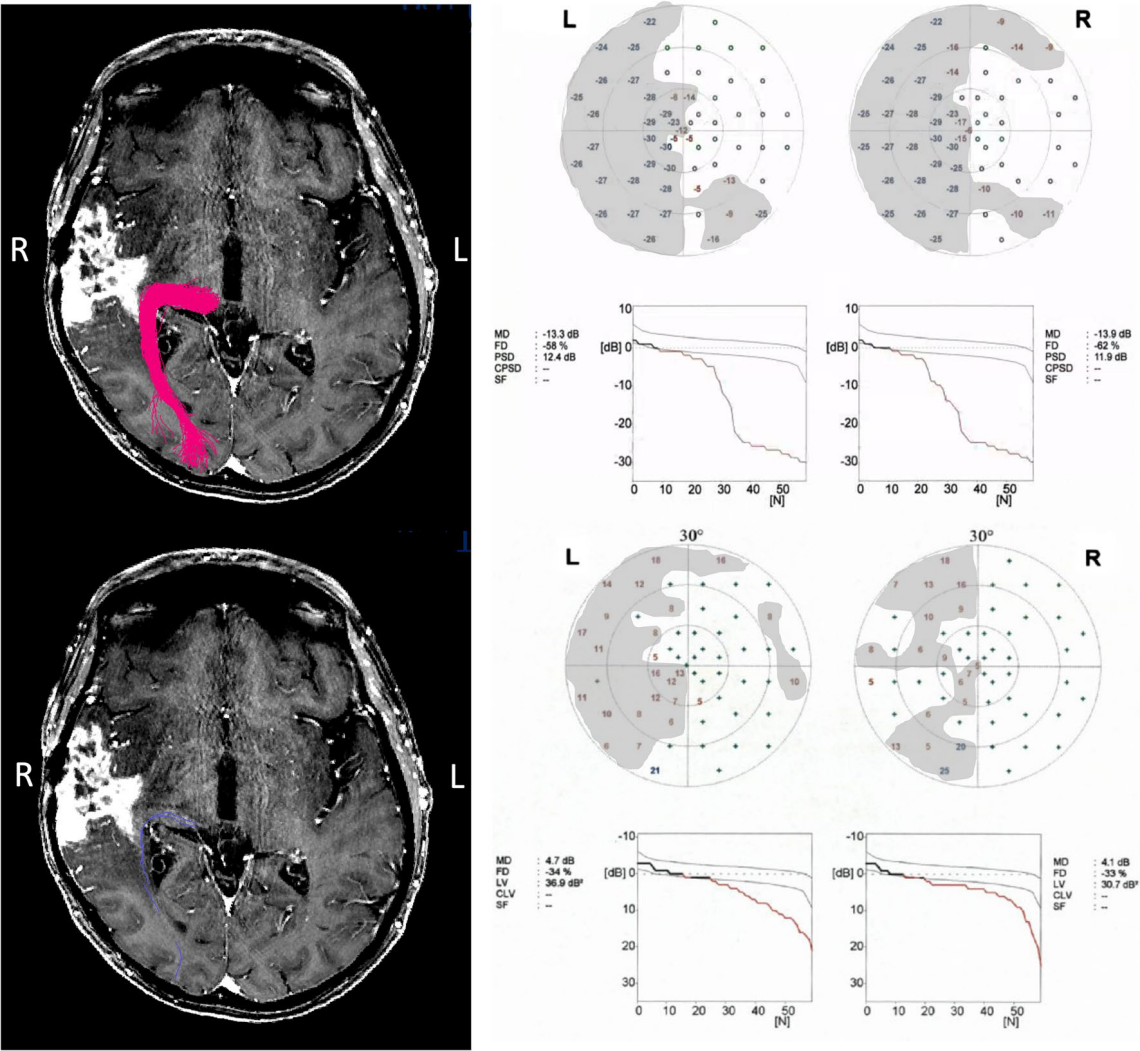
Case report for patient with improvement of visual field deficits and lesion in the medial temporal lobe, intrinsic to the OR based on QBI-FT, lateral according to DTI-FT. Left column, upper line: QBI-FT. Left column, lower line: DTI-FT. Right column, upper line: preoperative perimetry. Right column, lower line: postoperative perimetry. VFDs shaded in gray with defect depth < −5dB. MRI = magnetic resonance imaging; FT = fiber tractography; QBI = q-ball imaging; DTI = diffusion tensor imaging

A 58-year-old female patient was diagnosed with a temporal right-sided contrast-enhancing glioblastoma. Prior to surgical intervention, she manifested symptoms of left-sided hemianopsia. As illustrated in Fig. 2, both DTI and QBI-FT techniques were able to identify and trace the OR. Intriguingly, the OR appeared to be intrinsically associated with the tumor as suggested by QBI-FT, while a lateral attachment was indicated by DTI. Postoperatively, following a transtemporal approach, improvement in defect depth in both upper and lower quadrants was obtained.

#### Case 2

**Fig. 3:**
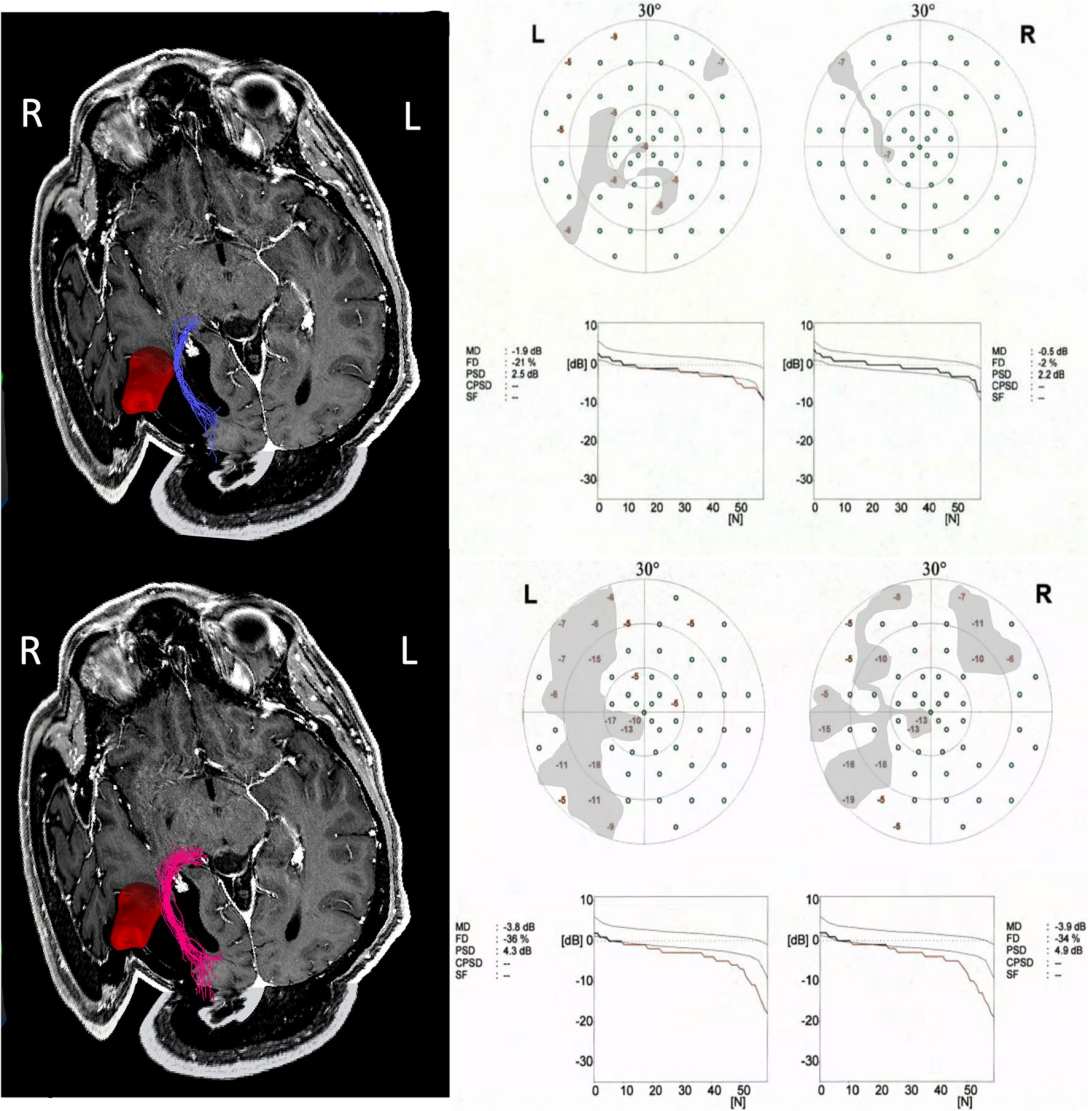
Case report for patient with recurrent temporodorsal glioma, lateral to the OR based on QBI-FT and DTI-FT. Lesion segmented in 3D red. Left column, upper line: DTI-FT. Left column, lower line: QBI-FT. Middle column: preoperative perimetry. Right column: postoperative perimetry. VFDs shaded in gray with defect depth < −5dB. MRI = magnetic resonance imaging; FT = fiber tractography; QBI = q-ball imaging; DTI = diffusion tensor imaging

A 43-year-old male patient was diagnosed with a temporodorsal, left-sided, non-contrast-enhancing astrocytoma. Preoperatively, he exhibited no VFDs. Both DTI and QBI-FT identified the OR extending from the LGN to the visual cortex, as depicted in Fig. 3. The OR was found to be laterally linked with the tumor, in accordance with the classification system of Faust et al., as suggested by both FT techniques. A transtemporal approach was chosen for the removal of the tumor. It is noteworthy that a postoperative deterioration was observed in the lower left quadrant, suggesting the potential injury to the fiber bundles, possibly as a direct consequence of extensive tumor resection.

## Discussion

The use of fiber tractography techniques to delineate the OR, especially the ML, might improve neurosurgical approaches for intracerebral lesions through prevention of potential injuries. To our knowledge, this is the first study to compare OR modeling using DTI- and QBI-based FT as well as assess pre- and postoperative VFDs using computerized perimetry. Compared with patients without preoperative VFDs, those with preoperative VFDs presented larger tumors and a smaller distance between the tumor and OR outline (nD-LOR) only in QBT-based FT analysis. Additionally, there was a trend of a negative correlation between the tumor size and nD-LOR only for QBT-based FT.

Using diffusion tensor imaging-based tractography, the authors in [7] delineated the optic pathway and compared with data from preoperative and postoperative visual fields of patients who underwent temporal lobectomy for pharmacoresistant temporal lobe epilepsy. Notably, the OR and ML could be identified in all the cases. Consistent with our findings, they observed a significant correlation between VFD and OR thermoresistant ML injury. Notably, they showed that intraoperative changes in fiber tracking results allowed valid and accurate prediction of postoperative VFD. Borius et al. reported a significant correlation between VFD and fiber damage during surgery for temporal lobe epilepsy [6]. Lilja et al. observed that the length of temporal lobe resection was positively correlated with the occurrence of postoperative VFD, as determined by deterministic and probabilistic fiber tractography models [17]. Contrastingly to the mentioned studies, we could visualize the OR in two cases only using QBI-based FT, while DTI-FT delivered no results. This could be attributed to the fact that gliomas cause white matter alterations due to their infiltrative behavior and thus impede reconstruction of the ML. Lu et al. reported that high-grade gliomas and metastatic tumors yielded significant changes in mean diffusivity and fractional anisotropy [18]. Specifically, the increase in extracellular bulk water may result in an increase of mean diffusivity, with the subsequent disorganized diffusion leading to a decrease in fractional anisotropy, impeding DTI-FT. Altogether, our results suggest particular superiority of HRFT over DTI-FT during brain tumor resection rather than in epilepsy surgery.

Mormina et al. analyzed the effects of high-grade gliomas on the OR and observed that DTI-based FT could not detect the extent to involvement of a specific white matter bundle in a tumor [21]. Contrastingly, HRFT allows more consistent detection of bundles involved in the course of the OR [21]. Our findings confirmed that QBI-FT might be a key tool in glioma surgery since it suggests a more precise delineation of the OR, particularly in cases with lesion-induced white matter alterations. Therefore, postoperative VFDs could potentially be predicted, as QBI-FT enables surgeons to model the operative region, thereby identifying potential injuries caused by either the tumor or the surgical approach. This insight could facilitate more effective patient management strategies.

Spherical harmonics are indeed employed in an array of neuroimaging applications beyond q-ball imaging (QBI), and their utility extends to methods like spherical deconvolution and analyses involving spherical ridgelets. These techniques have significantly advanced our ability to interpret the complex fiber architectures present within the brain, and their mention is pertinent to the comprehensive understanding of our study’s context within the field. In addition, the efficiency and capability of TractSeg to delineate the optic radiation (OR) within acceptable computation times is noteworthy. This software represents a significant progression in tractography, utilizing machine learning algorithms to streamline the processing of diffusion MRI data. While TractSeg was not utilized in our study, its inclusion in the discussion is valuable for readers and provides a benchmark for comparing the performance and efficiency of different tractography approaches. Moreover, we aim to explore emerging techniques in future research, such as free-water elimination methods, which may offer refined imaging insights, particularly for glioma characterization. However, these methods often require the acquisition of data at multiple *b* values, which can extend MRI scan times beyond typical clinical protocols. It is crucial to consider these practical aspects when discussing the translational potential of advanced imaging techniques. Our previous investigations have compared QBI with spherical deconvolution, and while the latter can offer in-depth analysis, it did not yield superior results for our specific clinical applications and was associated with increased postprocessing time. This finding underscores the importance of selecting imaging techniques that balance detail with practicality, particularly in a clinical setting. As we look to the future, the integration of big data analytics and machine learning in neuroimaging presents an exciting opportunity. Algorithms that learn and adapt could significantly enhance the accuracy of tractography and possibly reduce the time required for image processing.

## Anatomy of the optic radiation

To predict the anatomical relationship between the eloquent fiber tracts and mass lesions, it is important to elucidate anatomical landmarks as well as the pathological behavior of the lesions, including the infiltrative growth of gliomas. The OR starts in the LGN and forms a band that winds the inferior and posterior horns of the lateral ventricles, ending in the visual cortex or striate area with a three-bundle arrangement that comprises the anterior, central, and posterior bundles [28]. Notably, the anterior bundle runs anteriorly before making a sharp turn around the tip of the inferior horn, which is termed as Meyer’s loop, and passes posteriorly along the lateral wall of the ventricle, ultimately terminating at the lower lip of the calcarine fissure [23]. The central bundle initially runs laterally, then posteriorly, along the lateral wall of the ventricle, finally radiating into the calcarine cortex. Additionally, the posterior bundle directly runs in the posterior direction along the ventricular roof and converges at the upper lip of the calcarine fissure. The anterior bundle mainly preserves the superior quadrant; contrastingly, the central and inferior bundles preserve the posterior and inferior quadrants, respectively [28].

For presurgical planning, it is important to visualize the aforementioned structures in order to preserve them and prevent the occurrence or deterioration of VFD due to infiltration of the OR by intracerebral lesions. A major limitation of DTI-based FT is its inability to resolve complex intravoxel diffusion of kissing, crossing, or diverging fibers; alternatively, HRFT-models allow resolution of multiple intravoxel orientation. Kuhnt et al. reported that HARDI-based FT produced more convincing tractography results for gliomas near the OR than DTI-based FT [16]. This could be attributed to the fact that FT based on HARDI signals is less sensitive to factors such as tumor volume or size, peritumoral edema, and tumor morphology on MRI [16]. However, there have been no clinical evidence regarding the preoperative and postoperative visual acuity. Consistent with the findings by Kuhnt et al. [16], we determined that HRFT based on the q-ball model suggests higher-quality tractography results than DTI-based tractography, especially for larger gliomas when comparing VFDs and nD-LOR.

We used the classification system proposed by Faust et al. to determine the displacement of the OR with respect to tumor location [10] and included the localization for both DTI- and QBI-FT. We believe that this classification system might allow effective prediction of the postoperative occurrence or deterioration of VFDs based on the anatomical structure of the OR. Also, it seems the selected surgical approach significantly contributes to the incidence of postoperative VFDs.

The following inference can be drawn: Intrinsic localization through DTI-FT and QBI-FT has the potential to anticipate a progression of VFDs. It is hypothesized that QBI-FT may have a tendency towards overestimation, whereas DTI-FT in certain cases may not provide dependable results. Central defects seem to be associated with an occipital localization, also resected from an occipital approach, as observed in the two distinct cases. Harm to the anterior portion of the OR could exacerbate issues in the central areas of the retinae, as we have also observed for one lesion situated in the occipitomedian position [9].

### Surgical approach

In our study, we observed that patients with temporomesial lesions frequently developed visual field defects (VFD). This aligns with existing data, particularly from studies on temporomesial epilepsy, which indicate a propensity for VFD following surgery [25]. A plausible explanation for this phenomenon may lie in the surgical method employed, notably the transsylvian approach. Prior research focusing on temporal lobe epilepsy has established a significant correlation between this approach and postoperative VFD, including partial or complete quadrantanopia, with reported incidences ranging from 40 to 75% [12, 14, 25]. Typically, the transsylvian approach can adversely affect Meyer’s loop of the optic radiation, frequently leading to upper quadrantanopia. Partial quadrantanopia often manifests as a loss of vision in the superior, and occasionally inferior, parts of the visual field near the midline to the center. Larger lesions may extend this effect to the outer horizontal regions of the visual field. Additionally, there is a risk of vascular complications such as temporal ischemic lesions due to surgical retraction, potentially elevating the prevalence of VFD [19]. In contrast, the subtemporal approach may reduce the incidence of VFD postsurgery, as it tends to spare Meyer’s loop. This technique, aimed specifically at removing temporomesial lesions, avoids cutting through the temporal stem and enters the ventricle from its basolateral aspect [13, 25], offering an expansive view of the posterior hippocampal/mesiotemporal region. However, preserving basal veins, particularly the vein of Labbé, presents a challenge due to the retraction of the temporal lobe and the complexity of determining the optimal entry point on the inferior surface of the temporal lobe. Furthermore, resecting anterior structures like the uncus and amygdala proves more intricate with the subtemporal approach as compared to others. Despite these challenges, in glioma surgery, as opposed to epilepsy surgery, the primary objective is to achieve maximal safe resection to enhance patient prognosis and survival rates. This goal is often more feasible with the transsylvian approach. Advanced imaging techniques and fiber tracking are invaluable tools in mitigating such deficits. However, given the intricate anatomical considerations, completely preserving or avoiding these deficits can be exceedingly challenging.

For instance, damage to the anterior segment of the OR could potentially lead to a condition known as partial quadrant hemianopsia, a finding echoed in our current study involving patients with lesions located in the temporodorsal region. Using a transtemporal or subtemporal approach for resection of temporodorsal lesions lateral to the OR is also frequently associated with a deterioration of VFDs in our study. This might be due to the approach and pressure of retractors, but also an overestimated resection. We explain this with the surgeon’s attitude according to personal interviews: As opposed to pareses, VFDs are frequently rather accepted from surgeons for oncological patients. Understandingly, the oncological aspect preponderates in these cases.

QBI-based FT revealed that the distance between the tumor and OR tended to be smaller in patients with VFD than in patients without VFD. Additionally, QBI-based FT analysis revealed a negative correlation between tumor volume and its distance to the OR; therefore, QBI-FT might presumably predict VFDs better than DTI-FT, in cases of lager tumors and small nD-LOR. Taken together, QBI-based FT seems to provide more precise remodeling of the OR despite tumor-induced alterations. However, its clinical practicability is limited by its longer postprocessing time. In this way, QBI-FT cannot yet be used based on intraoperatively obtained MRI sequences.

## Limitations

The main strength of this study is that it is the first to compare conventional DTI- with QBI-based FT in presurgical planning for patients with lesions near the OR. However, this study has several limitations. First, a relatively small cohort of patients was included so far. Nonetheless, given the anecdotal evidence in this field, our findings provide a real-world picture of the feasibility of both techniques and might help to predict VFDs after tumor resection in such deterrent areas. Second, there could be observer variability due to manually drawn ROIs according to clinical knowledge. Notably, we previously reported excellent intrarater variability for the reproducibility of both techniques (DTI and QBI). A significant clinical drawback remains that QBI-FT is not yet implemented in the intraoperatively used navigation system. In this way, also a direct comparison to frequently intraoperatively used techniques like the multitensor model was not possible. For ongoing studies, other HRFT models will be considered, furthermore kinetic perimetry as offered by the original Goldmann perimetry. The current sample size, while providing valuable initial insights, does not yet allow for the establishment of universally applicable distance thresholds that could reliably indicate risk levels in neurosurgical planning. We recognize that establishing such thresholds would significantly enhance the utility of our findings in clinical practice. A larger and more diverse patient sample would provide the statistical power necessary to validate these thresholds, taking into account the multifactorial nature of brain lesions and their impact on surrounding neural structures.

## Conclusions

Our findings suggest that QBI-based tractography provides advantageous tractography results over DTI-FT in patients with lesions near the OR to prevent intraoperative damage. Additionally, the occurrence of postoperative VFD correlates with the location of the lesion in relation to the OR based on FT reconstructions. Notably, QBI-FT could solely reproduce the OR in selected cases; however, its clinical practicability is hampered by its extended postprocessing time.

## Data Availability

The datasets generated during and/or analyzed during the current study are available from the corresponding author on reasonable request.
